# Chromoblastomycosis of the face in solid organ transplant patient

**DOI:** 10.1016/j.jdcr.2022.11.004

**Published:** 2022-11-09

**Authors:** Stephanie Matthews, Bao Vincent K. Ho, Sarah Pourakbar, Atieh Jibbe

**Affiliations:** a Division of Dermatology, University of Kansas Medical Center, Kansas City, Kansas; b School of Medicine, University of Kansas Medical Center, Kansas City, Kansas

**Keywords:** chromoblastomycosis, dermatology, fungal infection, immunosuppression, infectious disease, tropical dermatology

## Introduction

Dematiaceous fungi are a distinct group of pathogens that can cause cutaneous or systemic diseases, particularly in states of immunosuppression, such as solid organ transplantation.^1^ Phaeohyphomycosis, mycetoma, and chromoblastomycosis are infectious syndromes caused by various melanin-pigmented dematiaceous fungi and are distinguished by specific histopathologic findings.^1^^,^^2^ The diagnosis of chromoblastomycosis is often delayed because of clinical similarities with other dermatologic diseases, such as sporotrichosis, verrucous tuberculosis, sarcoidosis, and cutaneous malignancies.^3^ Cutaneous manifestations consist of nodules, plaques, and verrucous lesions most commonly affecting the lower portions of the limbs due to inoculation from the soil and decaying vegetation in tropical countries.^3^ It rarely presents as a localized infection in the head and neck and has yet to be documented as being caused by *Phaeoacremonium* species^4^; we present an uncommon case of chromoblastomycosis overlying the mandible in a patient residing in the United States.

## Case report

**Fig 1:**
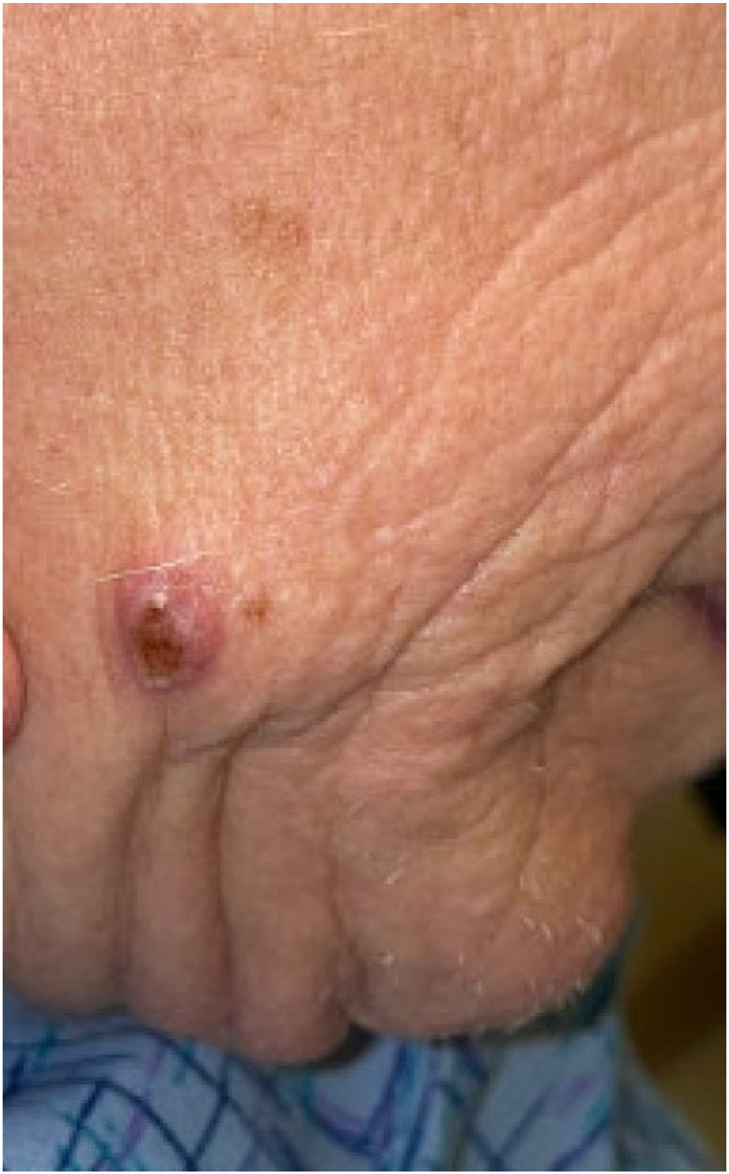
Clinical image of chromoblastomycosis infection of the jawline. Initial presentation of an excoriated papule on the right jawline. Enlarged crateriform nodule at the same site of the initial lesion 8 months later.

A 62-year-old woman receiving immunosuppressive therapy with a history of orthotopic cardiac transplant presented with a slowly growing, painful, scaly, erythematous nodule on the right side of the jawline. The patient first noticed a small, round, and excoriated pink papule 8 months prior (Fig 1). The patient did a trial of over-the-counter topical antihistamines and antibiotic ointments, which had no clinical effects. The lesion continued to grow in size and darken in color, prompting the patient to seek dermatologic evaluation.

Physical examination revealed a 2.7-cm crateriform erythematous nodule overlying the right side of the mandible. Differential diagnosis included keratoacanthoma; squamous cell, Merkel cell, and metastatic carcinomas; prurigo nodule; molluscum contagiosum; and deep fungal infection (Fig 1). The patient denied having a history of significant sun exposure or a prior history of skin cancer. Medical history was significant for cardiac transplantation and immunosuppressive therapy with mycophenolate sodium, tacrolimus, and prednisone, which she received 1 month after she first noticed the lesion. She could not recall any trauma to the head or neck region and denied experiencing fever, chills, unexpected weight loss, coughing, or shortness of breath. The remaining skin examination was negative for similar lesions.

**Fig 2:**
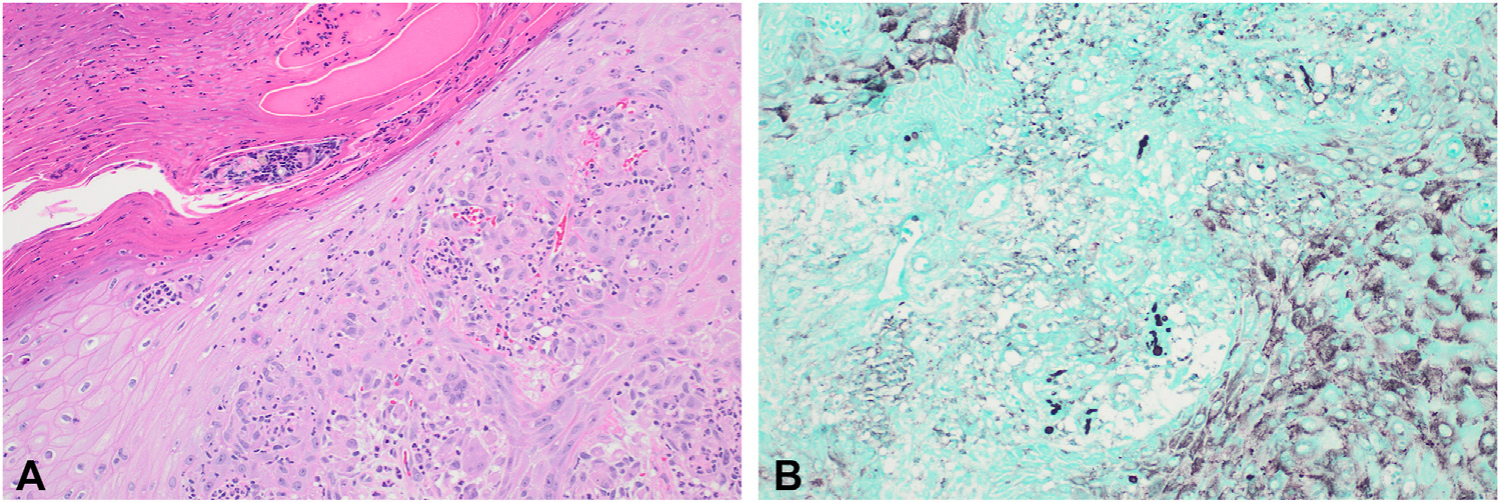
Histopathology of shave biopsy. **A,** Pseudoepitheliomatous hyperplasia with suppurative collections of neutrophils and fungal organisms. **B,** Melanized fungal structures and thick-walled sclerotic cells (medlar bodies) consistent with the diagnosis of chromoblastomycosis (**A,** Hematoxylin-eosin stain; **B,** Gomori methenamine silver stain; original magnifications: **A,** ×20; **B,** ×20.)

A shave biopsy was performed, the results of which revealed pseudoepitheliomatous hyperplasia with suppurative collections of neutrophils and fungal organisms suggestive of a deep fungal infection (Fig 2, *A*). Special staining with Gomori methenamine silver stain (Fig 2, *B*) and Fontana-Masson stain (not shown) revealed characteristic melanized fungal structures and thick-walled sclerotic cells (medlar bodies) consistent with the diagnosis of chromoblastomycosis. Blood cultures were negative for fungal organisms. Serum antibodies, antigens, and urine antigens for blastomycosis were negative.

Additional detailed history from the patient revealed an extensive travel history to multiple countries in her lifetime, most recently to Zambia (4 times within the preceding 5 years), where she worked as a missionary, planting crops on rural farms and volunteering in orphanages for several weeks in unsanitary conditions. The patient was referred to plastic surgery for excision and layered closure of the skin lesion, during which a tissue culture was obtained. The patient was also referred to infectious disease and started on oral itraconazole (400 mg/d). Tissue culture grew *Phaeoacremonium* species, and the antifungal therapy was subsequently switched from itraconazole to oral voriconazole (600 mg/d) due to increased susceptibility. She completed 8 months of antifungal therapy and did not have evidence of recurrence 1 year following surgical excision.

## Discussion

Chromoblastomycosis is a cutaneous infection caused by dematiaceous fungi endemic to Africa and Latin America.^4^, ^5^, ^6^ Causative fungi reside in the soil and inoculate the feet and legs through trauma. For this reason, most infections occur in the lower extremities.^4^^,^^6^ Lesions first begin as a papule and slowly develop into a scaly, erythematous, verrucous plaque or nodule similar in appearance to the head of a cauliflower.^4^, ^5^, ^6^ In skin of color, the lesions can appear violaceous instead of the pink/red appearance present in lighter skin types.^6^ If left untreated, complications, such as secondary bacterial infection, squamous cell carcinoma, tendon or joint invasion, lymphatic spread, and elephantiasis can occur.^5^^,^^6^

The classification and nomenclature of pigmented cutaneous fungal infections (phaeohyphomycosis, eumycetoma, and chromoblastomycosis) can be confusing.^7^ The infections are not differentiated by the causative organism but rather by their appearance on microscopy. Chromoblastomycosis is characterized by the presence of muriform bodies: brownish, round sclerotic spores with longitudinal and transverse cross walls.^3^^,^^5^^,^^6^ Although *Phaeoacremonium* species often cause phaeohyphomycosis, the presence of muriform bodies confirms the diagnosis of chromoblastomycosis.^4^ To our knowledge, chromoblastomycosis caused by the *Phaeoacremonium* species has not yet been reported.

Immunosuppression, namely solid organ transplantation, greatly increases the risk of *Phaeoacremonium* infection. The most recent systematic review regarding infected patients shows that 31 of 42 of those infected were immunosuppressed, and 17 of 31 had received a solid organ transplant.^8^ Additionally, patients taking oral tacrolimus experience more frequent, severe, and treatment-resistant fungal infections.^9^ Travel to endemic areas increases risk of exposure, illustrating the importance of obtaining an extensive travel history from immunosuppressed patients with unusual chronic lesions. A swift diagnosis is key for the prevention of disseminated infections.^8^ Further investigation is needed to assess the associated risks of tacrolimus use and fungal infection and whether the use of alternative immunosuppressive agents is appropriate for patients with a travel history to endemic locations.

Recommendations for effective treatment of cutaneous *Phaeoacremonium* lesions are lacking.^8^ Chemotherapy is currently used to treat progressive stages of infection.^3^ Antifungals terbinafine (250-500 mg/d) and itraconazole (300-400 mg/d) for 6 to12 months are most often used; however, success with flucytosine, amphotericin, and voriconazole has been reported.^6^ Antifungals can be combined with 5-fluorocytosine for cases refractory to antifungal treatment alone.^5^ Treatment options for direct removal include surgical excision, curettage, cryotherapy, CO_2_ laser photocoagulation, and electrodessication.^3^^,^^6^

This patient’s specific travel history, cardiac transplant, and immunosuppression increased her risk of dematiaceous fungal infection. The consideration of fungal causes for suspicious lesions is becoming more important for the modern clinician as the prevalence of immunocompromised patients continues to rise.^2^ Although uncommon in regions outside of endemic areas, the diagnosis of chromoblastomycosis should be considered when evaluating chronic lesions in immunosuppressed patients with a travel history to endemic regions.

## Conflicts of interest

None disclosed.
